# Surgical Treatment of Juvenile Hip Chondroblastoma Using Mosaicplasty: A Case Report

**DOI:** 10.3390/life16050752

**Published:** 2026-05-01

**Authors:** György Márk Hangody, László Rudolf Hangody, János Kiss, Miklós Attila Keszég, Gyula Ferenc Szőcs, László Hangody

**Affiliations:** 1 Department of Orthopaedics and Traumatology, Uzsoki Hospital, 1145 Budapest, Hungary; 2 Department of Orthopaedics, Semmelweis University, 1085 Budapest, Hungary

**Keywords:** chondroblastoma, osteochondral autograft transplantation, mosaicplasty of the hip

## Abstract

Chondroblastoma is a generally benign tumor occurring at a young age; however, its location near a joint and its tendency to recur make the treatment particularly challenging. This is especially true in the case of its occurrence in the hip joint. Surgical removal—curettage—is the primary method, but the remaining defect can be filled with several methods depending on the size of the tumor. The approach to the lesion is another difficulty. There are several available options, but due to the characteristics of the blood supply to the joint, this is a significant risk. In our case, we used an open autologous osteochondral graft transplantation (mosaicplasty) to treat juvenile hip chondroblastoma in a young female patient, for which the ipsilateral knee joint served as the donor area. The patient was followed up for 3 years after surgery, and, in addition to physical examinations, numerous imaging studies were performed to exclude local recurrence or avascular necrosis in the femoral head and to ensure that the congruence of the implanted osteochondral grafts was maintained.

## 1. Introduction

Chondroblastoma (CBL) is a rare, locally aggressive, benign bone tumor that originates from chondrocytes [1]. CBL accounts for only 1–2% of primary bone tumors and exhibits biological behavior with malignant tendencies [2], characterized by local invasion, metastatic potential, and a propensity for recurrence [3,4]. This tumor predominantly affects adolescents aged 12 to 20 years [5], with a male-to-female ratio of approximately 2:1 [6]. The distal femur, proximal humerus, and proximal tibia are the most common sites of occurrence [7]. However, the femoral head is an uncommon site, representing only 3.8–16.7% of cases [8,9]. Despite its slow growth, untreated CBL can lead to joint destruction, pain, and functional limitations [10]. Most patients complain of progressive pain that may mimic a chronic synovitis or other intra-articular pathological conditions. Radiographic findings are usually characteristic. This well-circumscribed lesion is usually centered in an epiphysis of a long bone; however, it also may be located in an apophysis, such as the greater trochanter or the greater tuberosity. Often, it has a surrounding rim of reactive bone, and 30% to 50% exhibit calcification on plain radiographs. Soft-tissue extension is extremely rare. In children, a well-circumscribed epiphyseal lesion that crosses an open growth plate is virtually diagnostic of chondroblastoma [7].

Histologically, chondroblastoma consists of sheets of chondroblasts, usually with a background of chondroid matrix. The cells are polygonal with distinct cytoplasmic outlines. Calcification is present and may surround individual cells, giving the classic “chicken-wire” appearance [7]. Aneurysmal bone cysts (ABC) are associated with chondroblastoma in 14% of cases [11]. The presence of a secondary ABC is often seen in CBL, and it was indicated as another risk factor for recurrence [12,13,14], although other series did not confirm these findings [15,16].

The classical treatment consists of extended curettage and bone grafting or placement of bone cement. Adequate curettage should always take precedence over sparing the growth plate [7]. The femoral head epiphysis presents a dilemma in terms of the best means of access, as the proximal femoral epiphysis is completely intracapsular and there is no access to the lesion without transgressing either the growth plate or the articular cartilage [11]. Due to the unique anatomical features of the femoral head, traditional curettage poses significant challenges, including difficult surgical access, incomplete lesion removal, and a high postoperative recurrence rate [17]. Moreover, improper surgical techniques may compromise the blood supply to the femoral head, increasing the risk of postoperative necrosis and dysfunction [18]. Therefore, optimizing surgical strategies and exploring minimally invasive treatment methods for femoral head chondroblastoma is of paramount importance [19].

Radiographs of the primary site should be obtained every 6 months for at least 3 years and annually thereafter. Recurrence occurs in 10% to 20% of patients and can be treated similarly to a primary lesion [7].

The appearance of chondroblastoma is usually accompanied by an osteochondral defect, which can be massive. Structural reconstruction of the damaged area can basically be managed by three methods: osteochondral autograft transplantation (mosaicplasty), osteochondral allograft transplantation [20], or endoprosthesis implantation. The latter—considering its last resort nature—should be avoided at a young age. Based on this consideration, for a biological reconstruction, either autologous or allogenic osteochondral transfer could serve as a practical alternative. Transplantation of a fresh osteochondral allograft can offer a benefit to avoid any kind of donor site morbidity, but the incorporation of the grafted tissue may require a longer period, and congruency problems may also compromise the success rate. Therefore, autologous osteochondral transfer may also be considered as a promising biological resurfacing option. Autologous osteochondral mosaicplasty for osteochondral lesions was introduced to clinical practice in 1992, and the first promising results were published by Hangody in 1994, and since then, its popularity has risen. The long-term survival of the transplanted chondrocytes and osteocytes has been demonstrated by histological studies [21,22,23].

The advantage of mosaicplasty is that by transplanting cylindrical osteochondral grafts, a hyalin-type surface can be created in the weight-bearing area of the joint, which is considered a biological solution with good long-term results [24]. However, its feasibility is limited due to the limited donor area (it is recommended for medium-sized defects, up to approximately 4 cm^2^ in size), and in the case of open growth plates, the epiphyseal growth plate may be damaged during graft harvesting and drilling the recipient side, thus causing growth disruption.

Mosaicplasty is a well established surgical procedure for the knee. However, there is little evidence that this method can also be used to treat osteochondral lesions in the hip. Hangody and Füles reported in 2003 that osteochondral transplantation was performed on six femoral heads. However, the specifics of the surgical process and the clinical outcome were not covered [25].

When the defect is in the hip joint, several factors make the procedure difficult. The joint is inherently difficult to open due to the blood supply characteristics of the capsule and can lead to circulatory disorders. In rare cases, the defect becomes accessible after capsule-sparing opening, but in most cases, the femoral head must be subluxated to reach the problematic area, which is also a compromising factor in terms of circulation [26].

Due to limited evidence and a lack of experience, mosaicplasty treatment of these lesions remains challenging, especially in young patients [27].

Based on the above considerations, in our case, we used mosaicplasty for the surgical treatment of a chondroblastoma in the femoral head of a 13-year-old girl, and the ipsilateral knee joint served as the donor area.

## 2. Case Presentation

**Figure 1:**
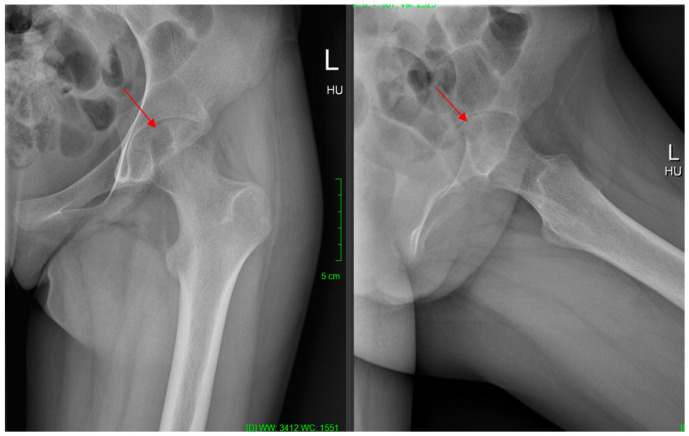
Preoperative 2-dimensional hip joint radiograph clearly showing the cystic lesion in the femoral head.

**Figure 2:**
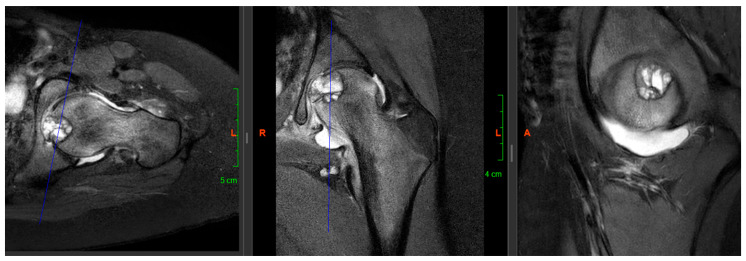
Preoperative MRI scan (transverse, coronal and axial sections).

A 13-year-old female patient (K.A.—born: 2009), who started having left knee pain in September 2021, but the physical and imaging examinations performed did not reveal any changes in the joint. The pain became constant in her knee, but in the beginning of May 2022, the patient reported rapidly increasing left hip pain. In June 2022, she had to use crutches to walk, and by July, she had developed significant limitation of movement, so she was re-examined physically and after that, X-ray, CT, and MRI scans were taken. The completed X-ray examination showed cystic thinning in the femoral head (Figure 1), which is why the other two imaging modalities were also necessary. On the latter, approximately 2 cm in diameter (~3–4 cm^2^), sharp-edged, perifocal edema-free lytic area was visualized in the left femoral head—subcortically—in the area of the epiphysis (Figure 2).

The patient arrived at our institute in August 2022 for consultation. The tall, extremely thin female patient walked with a mild limp using two elbow crutches. During physical examination, there was approx. 30 degrees of flexion and a slightly adducted contracture of the left hip. The extension deficit could be reduced to 20 degrees with passive stretching in a relaxed position. In order to surgically resolve the tumor lesion detailed above, we decided to expose the hip joint, then, after its subluxation, excochleate the lesion and perform mosaicplasty with osteochondral autograft harvested from the ipsilateral knee joint. The planned intervention took place at the end of August 2022.

**Figure 3:**
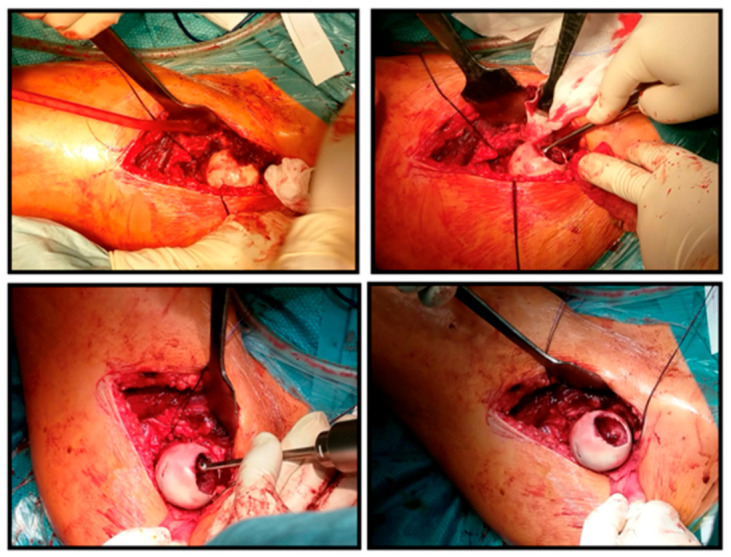
Surgical images of the tumor tissue after subluxation of the femoral head, its scraping with a Volkmann spoon, and then the cleaning of the tumor bed with a ball bur.

**Figure 4:**
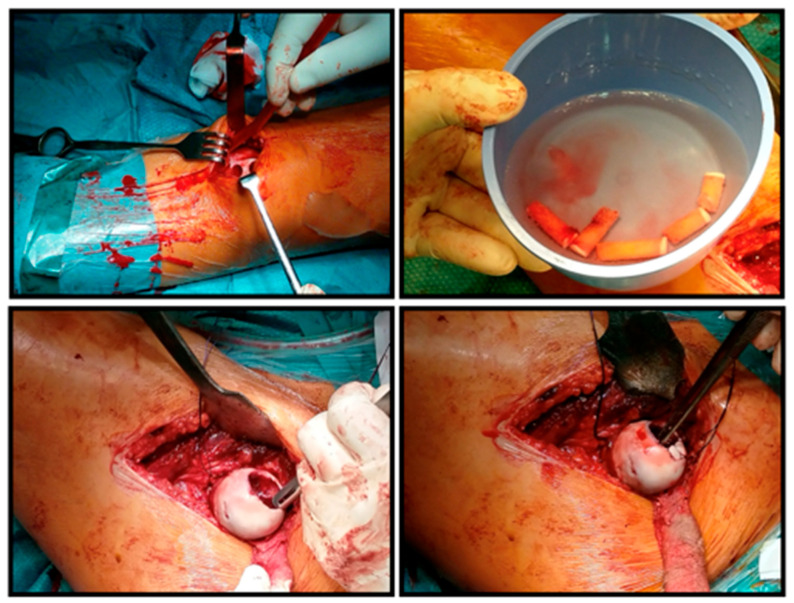
Surgical images of graft harvesting from the knee joint and the obtained 5 osteochondral grafts. The bottom 2 images of the montage show the creation of the drill channel on the recipient side and the implantation of the first 2 grafts.

The surgery was performed through an anterolateral approach, after which the joint capsule was reached between the tensor and sartorius muscles. The incision of the capsule was made in a minimalized T-shape so that the horizontal stem ran directly under the labrum and did not reach the capsule attachment distally, so that no bleeding was experienced from the joint capsule. As a next step, the femoral head was carefully subluxated and the fingertip-sized, red tumor tissue located in the center of the head was removed by excochleation and sent for histological examination (stored in formalin). The surface was excised up to the intact cartilage, the bone base was scraped, and the resulting 7–8 mm deep bone bed was refreshed with a ball burr (Figure 3). Then, from a mini arthrotomy, we obtained five cylindrical osteochondral grafts, 20 mm long and 8.5 mm in diameter, from the edges of the medial and lateral femoral condyles of the same knee joint, sparing the femoral head. After pre-drilling and conical expansion in the area of the defect on the femoral head, we performed mosaicplasty by inserting the grafts (Figure 4).

**Figure 5:**
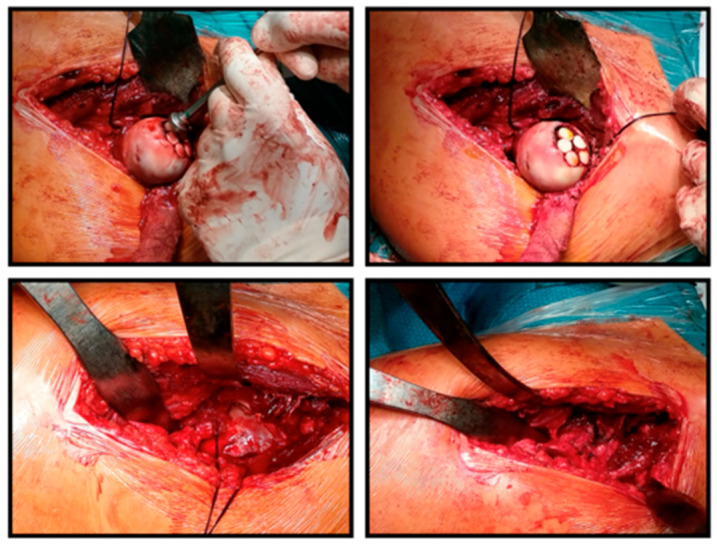
Surgical images of the implantation of additional grafts, their completion, and finally the closure of the joint capsule.

An important aspect of the implantation was the creation of short drill channels so that the physis was not damaged, and due to the curvature of the femoral head, the mutually supporting convergent implantation was a so-called arc-shaped arrangement. Then, we reduced the hip, reconstructed the joint capsule (Figure 5) and closed the wounds in layers.

The patient was completely unloaded for 4 weeks in the postoperative period, followed by a further 2 weeks of partial weight-bearing (approx.: 30 kg). During the histological examination, chondroblastoma with secondary aneurysmal bone cyst details was confirmed in two independent examination institutions.

**Figure 6:**
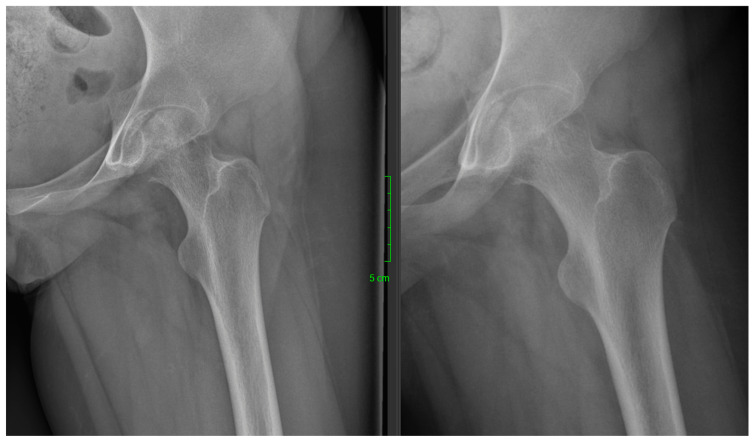
Postoperative AP hip joint radiographs taken at 6 and 12 months.

**Figure 7:**
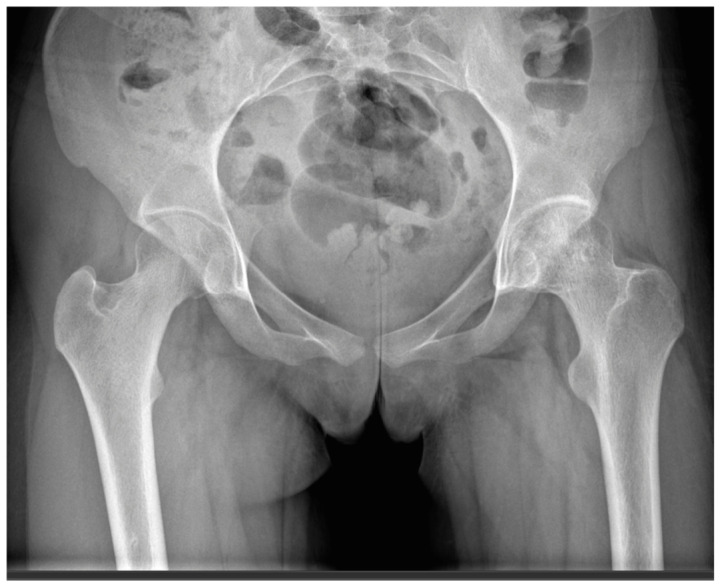
Postoperative AP pelvic radiograph taken at 3 years of age. The image shows preserved articular surface congruence. There are no signs of AVN.

**Figure 8:**
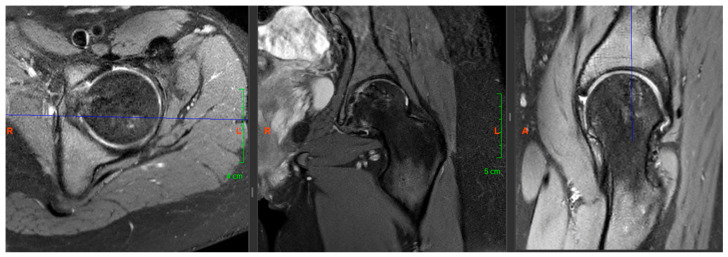
Postoperative MRI scan taken at 3 years of age, showing good joint congruence and proper integration of the grafts.

As for the radiological follow-up, a total of five x-rays were performed up to 1 year of age (at 6 weeks, 3-, 6-, 9- and 12 months), which showed adequate contour and good graft integration, and no necrosis was observed (Figure 6*)*. MRI scans were performed at 6 and 15 months. In the former, an inhomogeneous structure was still visible in accordance with the replacement (no demarcated necrosis), while the latter showed good graft integration and excellent contour in the load-bearing zone and minimal edema in the surrounding area. In addition, a CT scan was performed at 12 months. This imaging modality did not show graft necrosis or chondroblastoma recurrence. The patient was last followed up 3 years after surgery, at which time we performed X-rays and MRI scans again, which still showed an impeccable contour in the replaced area and no signs of necrosis (Figure 7 and Figure 8).

The patient’s previously restricted hip joint range of motion and limping improved significantly after the surgery, and he had no remarkable pain with hip movements. However, despite intensive physiotherapy sessions and local relaxation treatments, her rotational range of motion remained restricted, and a mild hip joint contracture of rotation and flexion and minor limping were recorded. The latter is due to an apparent limb length difference, one component of which is the pelvic asymmetry resulting from his scoliosis, and the other is the contracture of the operated hip.

Preoperatively, the patient presented with severe pain, contracture, and limited mobility requiring crutches, whereas postoperatively, significant clinical improvement was observed, including pain reduction, improved gait, and increased range of motion.

## 3. Discussion

The proximal femur is the third most common site affected by CBL after the proximal tibia and proximal humerus [28]. It represents one of the most challenging anatomical locations for the treatment of chondroblastoma because of the limited surgical access to the femoral head and the potential risk of vascular compromise [29]. In young patients with an open growth plate, where epiphyseal CBL is located very close to the physis and articular cartilage [15,28,30,31,32], there is a high risk of recurrence and delayed postoperative functional recovery. The anterior arthrotomy poses the risk of femoral head avascular necrosis [28,33]. Although extended intralesional curettage combined with bone grafting remains the most commonly used treatment method, several authors have highlighted the limitations of this approach when the lesion is located close to the articular cartilage or the growth plate.

In the present case, mosaicplasty was selected based on several specific clinical considerations. The defect size (~3–4 cm^2^), its subchondral location in a weight-bearing area, and the young age of the patient made biological reconstruction preferable. Alternative options such as curettage with bone grafting alone would not restore the articular cartilage surface, while osteochondral allograft transplantation may present challenges related to graft integration and availability. Therefore, autologous osteochondral transplantation was considered the most appropriate method to restore both the subchondral bone and cartilage surface in a single procedure.

Ramappa et al. reported that while intralesional curettage is generally effective, recurrence rates of chondroblastoma may range between 10 and 20%, particularly when lesions are located in epiphyseal regions such as the femoral head [31]. Similarly, Strong et al. analyzed the management of femoral head chondroblastoma and emphasized that the anatomical constraints of this region often complicate complete tumor removal and reconstruction of the articular surface [11].

In recent years, alternative techniques have been explored to address these limitations. Muratori et al. demonstrated that surgical outcomes may improve when structural reconstruction of the articular surface is considered after tumor removal, particularly in weight-bearing joints [9]. Reconstruction techniques that restore the osteochondral architecture may therefore reduce the risk of secondary degenerative joint changes.

Among the available biological reconstruction techniques, osteochondral autograft transplantation (mosaicplasty) offers several advantages. As demonstrated in the systematic review by Athanasiou et al., mosaicplasty provides viable hyaline cartilage coverage and stable press-fit fixation, allowing restoration of joint congruency and load distribution [27]. Although most clinical experience with mosaicplasty originates from knee surgery, increasing evidence suggests that the technique may also be applicable in selected hip joint lesions. Despite the favorable outcome observed in this case, mosaicplasty in the femoral head remains technically demanding. The procedure carries potential risks, including vascular compromise leading to avascular necrosis, limited surgical access, and donor-site morbidity. Furthermore, long-term durability remains uncertain, as most available evidence originates from knee applications rather than hip joint reconstructions.

A potential cause of failure is insufficient press-fit fixation of the transplanted grafts, which has been reported in the literature as a very rare technical complication. In our case, we performed a stepwise implantation in an arc-shaped configuration to minimize this risk. Based on the systematic review of hip mosaicplasty cases by Athanasiou et al. [27] and other long-term results published about knee mosaicplasty cases, donor-site morbidity may represent a limiting factor in the long-term clinical outcomes. The generally accepted upper limit of the defect size is 3–4 cm^2^, which is associated with an acceptable level of donor site morbidity [34].

In our case, the defect size remained within accepted indication criteria, and no donor-site-related complaints were observed during the 3-year follow-up period. Mosaicplasty was chosen due to the moderate size of the osteochondral defect and the young age of the patient. Compared with allograft reconstruction, autologous osteochondral transplantation avoids immunologic concerns and may promote faster biological integration. At the same time, the press-fit fixation, the convergent structural design of the grafts and the tight joint conditions ensure immediate mechanical stability.

The favorable clinical and radiological outcomes observed during the three-year follow-up period support the feasibility of this approach. No recurrence of the tumor or signs of avascular necrosis were detected, and the congruence of the reconstructed articular surface remained preserved.

Although this report represents a single case, it contributes additional evidence supporting mosaicplasty as a potential biological reconstruction method following curettage of femoral head chondroblastoma. Further clinical studies with larger patient cohorts and longer follow-up periods will be necessary to determine the long-term durability of this technique. This is a potential biological solution for a rare location and type of osteochondral lesion, for which the literature is quite lacking in terms of comparative results. Therefore, there are no satisfactory data regarding long-term durability, so we rely on the experience gained with osteochondral defects of the knee joint [35]. A major limitation of this report is the absence of standardized functional outcome scores (e.g., Harris Hip Score or HOOS), which limits objective comparison with existing literature. Although clinical improvement was evident based on physical examination and patient-reported symptoms, this limitation should be considered when interpreting the results.

Due to the rarity of femoral head chondroblastoma and the limited number of reported reconstructive techniques, individual case reports continue to provide clinically relevant insights despite their inherent limitations.

## 4. Conclusions

There are several surgical options for treating chondroblastoma. The location and extent of the lesion, the patient’s age, and the involvement of the joint surface play a decisive role in choosing the type of intervention. In the case of hip joint defects, the blood supply to the joint is also a particularly difficult factor. In the case detailed above, taking all these factors into account, we used mosaicplasty to treat the defect in the femoral head, with which we gained good experience during a 3-year follow-up.

Mosaicplasty may represent a feasible biological resurfacing option in carefully selected cases of femoral head chondroblastoma, particularly in young patients with moderate-sized osteochondral defects. However, given the limitations of a single-case report and the lack of long-term comparative data, further studies with larger cohorts and long-term follow-up are required to confirm its broader applicability and durability.

## Data Availability

The data supporting the findings of this study are available from the corresponding author upon reasonable request.
